# Construction and validation of a mobile technology to support mothers of preterm newborns

**DOI:** 10.1590/0034-7167-2024-0336

**Published:** 2025-09-01

**Authors:** Thaís Emanuele da Conceição, Maria Helena do Nascimento Souza, Rafael Braga Esteves, Patrícia Lima Pereira Peres, Donatella Valente, Giovanni Galeoto, Antonella Nespoli

**Affiliations:** I Universidade Federal do Rio de Janeiro. Rio de Janeiro, Rio de Janeiro, Brazil; II Universidade Estadual do Rio de Janeiro. Rio de Janeiro, Rio de Janeiro, Brazil; III Sapienza Università di Roma. Lácio, Roma, Itália; IV Universidade de Milano-Bicocca. Milão, Lombardia, Itália

**Keywords:** Mobile Applications; Infant Premature; Patient Discharge; Home Nursing; Nursing Care, Aplicaciones Móviles, Recién Nascido Prematuro, Alta del Paciente, Atención Domiciliaria de Salud, Atención de Enfermería

## Abstract

**Objectives:**

to develop and validate the content of a mobile technology as a tool for the care of preterm newborns at home.

**Methods:**

a methodological study of a descriptive, qualitative, and quantitative nature, involving the development of an application, which was designed and validated through three stages: a literature review and analysis of interviews with mothers; application development; and content validation by panel of experts.

**Results:**

through an integrative literature review and analysis of interviews with 10 mothers, the topics incorporated into the technology were identified and subsequently validated by 14 experts, achieving a content validity index and coefficient above 0.80 across the 17 evaluated items, with an internal consistency of α=92.6.

**Conclusions:**

the development of the application for mothers of preterm newborns demonstrated a high degree of agreement among the expert panel, confirming that the developed content is valid and serves as an additional care technology for nurses.

## RESUMEN

**Objetivos:**

construir y validar el contenido de una tecnología móvil como herramienta tecnológica para el cuidado del recién nacido pretérmino en el domicilio.

**Métodos:**

estudio metodológico de carácter descriptivo, cualitativo y cuantitativo, que consistió en la elaboración de una aplicación, construida y validada a partir de tres etapas: revisión bibliográfica y análisis de entrevistas con las madres; desarrollo de la aplicación; y validación del contenido por panel de especialistas.

**Resultados:**

a través de la revisión integrativa de la literatura y el análisis de entrevistas con 10 madres, fue posible identificar los temas incorporados en la tecnología, los cuales fueron validados por 14 especialistas, con un índice y coeficiente de validez de contenido superiores a 0,80 en los 17 ítems evaluados y una consistencia interna de α=92,6.

**Conclusiones:**

la construcción de la aplicación para madres de recién nacidos prematuros mostró un alto grado de concordancia por parte del panel de especialistas, demostrando que el contenido desarrollado es válido y representa una nueva tecnología de cuidado para el profesional de enfermería.

## INTRODUCTION

The long journey that Brazil has undertaken since the 1980s has demonstrated numerous scientific and technological advances in neonatal health, significantly contributing to the reduction of infant and neonatal mortality. However, prematurity, defined as birth before 37 weeks of gestational age, remains a global public health issue^(1)^.

Although preventable, approximately 12% of births in Brazil are estimated to be preterm, a high rate compared to developed countries^(1)^. Furthermore, the current neonatal mortality rate in Brazil is 7.87, exceeding the national target set by the Sustainable Development Goals (SDGs) for 2030, which aims to reduce neonatal deaths to fewer than 5.0 per 1,000 live births in order to mitigate population vulnerabilities^(2)^.

Indeed, children born prematurely require comprehensive care. In this regard, the role of primary healthcare professionals, particularly those in family health teams, is crucial in coordinating holistic care for families within their service area, as well as in providing guidance on daily newborn care^(3,4)^.

Moreover, considering Brazil’s unique healthcare landscape, primary healthcare is positioned as a key strategy for ensuring comprehensive care for the neonatal population and their families, addressing challenges related to the implementation of the SDGs and the execution of the 2030 Agenda. The principle of “Leaving No One Behind” presents a significant challenge in achieving comprehensive care, regardless of social conditions^(2)^.

Evidently, the experience of preterm birth can disrupt families’ expectations formed throughout pregnancy due to the hospitalization of the newborn, the discharge process, and the transition to home care, often accompanied by distress and difficulties^(5)^. Thus, beyond the assistance provided across various levels of the healthcare network, the use of technologies, such as mobile applications, enhances the effectiveness of health education and serves as an essential tool for ensuring continuity of care. These technologies facilitate access to guidance provided from the hospital stay through discharge, promoting technology-mediated care^(6)^.

Additionally, technology has become a powerful ally in disseminating accurate information, enabling healthcare professionals, particularly nurses, to integrate validated tools into their practice and make them accessible to patients whenever needed. This fosters a closer connection between users of the Unified Health System (SUS in Portuguese) and the technological environment^(6,7)^.

Given the magnitude of this issue and the evident lack of neonatal nursing applications available in the digital environment for integration into public health programs, this study highlights the importance of research on the use of technology in the home care of preterm newborns. It also emphasizes the need for further studies to assess the relevance and adequacy of a new technological tool^(8)^.

## OBJECTIVES

To develop and validate the content of a mobile technology as a tool for the care of preterm newborns at home.

## METHODS

### Ethical aspects

This study adhered to Resolution No. 466/12 and Circular Letter No. 1 of March 3, 2021, and was approved on April 13, 2022, by the Research Ethics Committee of the Federal University of the State of Rio de Janeiro – Anna Nery School of Nursing. Participants were required to provide informed consent via the Informed Consent Form (ICF), which was sent electronically through the Google Forms platform and/or signed in person.

### Study type

This is a methodological, descriptive study that involved the development and validation of the content of an application designed for use in the home care of preterm newborns. The study was conducted based on the Consolidated Criteria for Reporting Qualitative Research (COREQ), following the guidelines of the EQUATOR Network, which standardizes the reporting of qualitative studies^(9)^.

### Study setting

The study was conducted in a Neonatal Intensive Care Unit (NICU) of a maternity hospital specializing in the care of high-risk pregnant women, located in the city of Rio de Janeiro.

### Population and selection criteria

For the first phase of the study, which involved defining the theoretical content to be incorporated into the application, the researcher included ten participants—mothers of preterm newborns—selected through convenience sampling. The inclusion criterion was being a mother of a preterm newborn who had been born and admitted to the NICU. Exclusion criteria included mothers under 18 years of age and mothers of newborns with congenital malformations or those requiring invasive devices and specialized home care after hospital discharge.

Following the second phase, which involved developing the application prototype, participants were selected to validate the application. In this third phase, fourteen nurses were chosen as expert evaluators.

The identification of experts was conducted through a search on the Lattes Platform of the National Council for Scientific and Technological Development (CNPq in Portuguese) and via the non-probabilistic snowball sampling technique, in which initial participants refer additional participants^(7)^.

For the inclusion of expert nurses, the study adopted the criteria established by Jasper (1994)^(10)^, which required professionals to meet at least two of the following qualifications: Possess skills or knowledge acquired through experience; Have skills or knowledge that make them an authority on the subject; Have passed a specific test to identify experts.

As a result, 27 (twenty-seven) specialists were identified, all of whom were nurses with specialization and professional experience in neonatology and expertise in the care of preterm newborns. Of these, 14 (fourteen) responded to the survey via an electronic questionnaire sent by email after telephone contact. The sample was deemed appropriate for content validation studies^(11)^.

### Study Protocol

The study was developed in three phases: a literature review and analysis of interviews with mothers, application development, and content validation by a panel of experts, following the methodology established by the User-Centered Interaction Design model. This model serves as a structural framework that requires the participation of the end user in the creation phase of computerized systems, such as mobile applications^(12)^.

Thus, the study phases were as follows:

### Phase 1 – Literature review and analysis of interviews with mothers

For the first phase, a literature review was conducted in six databases: Exerpta Medica Database (EMBASE), Medical Literature Analysis and Retrieval System Online (Medline) via PubMed, Web of Science via the CAPES Journal Portal, Literatura Latino-Americana em Ciências da Saúde (LILACS), Scientific Electronic Library Online (SciELO), and Cochrane Library. Searches were conducted in these electronic databases between January and May 2022, guided by the following research question: “What are the scientific publications from 2012 to 2021 regarding maternal concerns in caring for preterm newborns at home?”.

The search strategies included controlled descriptors retrieved from the *Descritores em Ciências da Saúde* (DeCS) and Medical Subject Headings (MeSH) via PubMed, in English and their synonyms, as well as uncontrolled descriptors, all connected by the Boolean operators AND and OR. Additionally, truncation symbols were used depending on the specific characteristics of each database.

Within this first phase of the study, semi-structured interviews were also conducted with the following open-ended questions: “What are your concerns regarding the care of your child?”; “Of all the aspects of preterm newborn care, what do you fear most when doing it alone?”; “What information would you like to have available when at home?”; and “Who are the people in your support network?”.

The interviews with mothers took place between August and September 2022 in a designated private space within the hospital unit where the newborns were monitored after NICU discharge. Each interview lasted an average of ten minutes. The study included ten mothers of preterm newborns, aged 20 to 44 years, who were still hospitalized in the NICU. The objective was to complement the research and practically identify the challenges faced by caregivers.

Following the literature review, which included 21 articles published in journals—most of which were classified as level VI evidence—and after conducting maternal interviews, which were completed once data saturation was reached, fully transcribed, and analyzed in detail, it was found that the main concerns were related to breastfeeding, hygiene care, colic, sunbathing, recognition of warning signs, and the Kangaroo Method. Consequently, six major themes were identified to guide the development of the application for home-based care of preterm newborns: Kangaroo Method, Breastfeeding, Sleep and Rest, Hygiene Care, Warning Signs, and Neuropsychomotor Stimulation.

### Phase 2 – Application Development

Based on the topics identified for the conceptualization and theoretical construction of the technology, a mobile application model was designed and developed online using the free website Canva.com, incorporating public domain images created in accordance with the previous phases and following the established methodological model.

During the conceptualization of the material, the initial screen, following the cover screen, consisted of a logo featuring footprints representing those of newborns and the name *Meu RN Prematuro* in purple, as this color is used to symbolize World Prematurity Day^(13)^.

The next screen included fields for entering identification data such as the baby’s name, date of birth, corrected gestational age, weight, and hospital discharge date. These fields are to be completed by the mother or the unit’s nursing professional at the time of the newborn’s discharge to home care.

The item “corrected gestational age” was included due to the impact of prematurity on neonatal growth and development. This feature aims to support the continuity of care within the primary healthcare network, considering that specific evaluation charts have been developed for preterm newborns to be used during follow-up consultations.

The following screen was designed to promote bonding between the newborn and their family, allowing the insertion of photographic records taken during hospitalization according to the preferences of the caregiver and other involved individuals.

### Phase 3 – Content Validation by a Panel of Experts

A total of 14 (fourteen) participants responded to the study for the purpose of validating the application’s content, all of whom were female, with ages ranging from 25 to 60 years. The majority were within the 36 to 45-year age range (64.3%).

The expert panel members resided and worked professionally in the state of Rio de Janeiro (RJ) within the public healthcare system and had extensive professional experience in hospitals specializing in preterm neonatal care. Of these, 92.9% had five or more years of professional practice.

To analyze the results, a database was created in Excel, where each question from the structured instrument for application validation—regarding content relevance, written language, user appeal, and topic pertinence—was assigned a numerical code based on five response options: “strongly disagree (1),” “disagree (2),” “neither agree nor disagree (3),” “agree (4),” and “strongly agree (5).”

For the validity and reliability analysis of the items contained in the application, the experts evaluated the criteria using the Statistical Package for the Social Sciences (SPSS) software, calculating the Content Validity Index (CVI) and the Content Validity Coefficient (CVC) for each item. The validation criterion adopted was a value greater than 0.80^(11)^, considering the average of the “4” and “5” responses assigned by the expert panel.

To verify internal consistency, Cronbach’s alpha was calculated, with values between 0.70 and 0.95 indicating strong correlation. Additionally, an exact binomial distribution test was performed, adopting a significance criterion of p > 0.05.

## RESULTS

The results of the first phase supported the development of the application’s content.

Based on the systematic literature review, which analyzed 21 articles published between 2012 and 2021, the primary concerns of mothers regarding the home care of preterm newborns were identified as breastfeeding, hygiene, sunbathing, infant colic, clinical changes, temperature regulation, and the Kangaroo Method^(14)^.

This phase also considered the results of interviews conducted with ten mothers of children born between the 25th and 36th weeks of gestation. These mothers were between 20 and 44 years old; the majority were single (6), had completed elementary school (6), and did not have paid employment outside the home (6).

Regarding uncertainties related to preterm newborn care, mothers reported concerns about breastfeeding, hygiene care, formula feeding, identifying signs of abnormalities, sleep, and rest, as illustrated in the following excerpts:

*My only concern at home, I think, will be about feeding. Since her stomach is smaller, what is the right time to breastfeed?”.* (Mother ٠٦)
*Will I be able to bathe this child?* [...] *But I want to feel more confident when bathing.* (Mother 03)
*I see that she is having difficulty sucking the bottle; I notice that it takes her a long time.* (Mother 08)
*He needed to use oxygen, so at home, I will have to pay more attention to that. If he feels unwell, I’m not sure I would be able to identify it. I don’t think so.* (Mother 05)
*My concern with him is about breathing at home.* [...] *Breathing really worries me. And vomiting—him bringing everything up.* (Mother 09)
*I have never been a mother. I have doubts about bathing and how to put him to sleep.* (Mother 10)

With the results of the literature review and the data obtained from the interviews, it was possible to structure the content of the application and proceed to validation by specialists.

Thus, regarding the structure and availability of the application’s themes, an organizational structure was implemented to ensure coherence in the content. In other words, the sequence of information available on the main screen will enable users to connect themes, thereby offering a structured approach to newborn care.

Additionally, all content was structured in accordance with the grammatical rules of the Portuguese language in effect in the national territory and reviewed by a specialist in the field before being inserted and made available in the application. This ensured coherence, appropriate language use, and compliance with grammatical norms. Therefore, semantic evaluation was not included in this phase of the study.

Regarding the profile of the expert panel participants, it was found that they specialized in neonatology (42.9%), followed by those with a master’s degree (28.6%) and a doctorate (28.6%).

Data related to the characterization of the expert panel are presented in Table 1.

**Table 1:** Profile of the expert panel, Rio de Janeiro, 2023, (N=14)

Characteristics	n	%
Age (in years)		
25 – 35	03	21.4
36 – 45	09	64.3
46 – 60	02	14.3
Academic Qualification		
Specialization	06	42.9
Master’s degree	04	28.6
Doctorate	04	28.6
Years of Professional Experience in Neonatal Nursing		
3 to 4	01	7.1
5 or more	13	92.9

Regarding the items evaluated by the specialists, the average I-CVI was 0.96, and the average CVC was 0.91 (Table 2), reflecting excellent agreement ratings concerning the content and appearance of the final version of the proposed application, which demonstrated an internal consistency of α = 92.6.

**Table 2:** Evaluation by specialists regarding the clarity, relevance, and appearance of the application, according to the calculation of the Content Validity Index and the Content Validity Coefficient, Rio de Janeiro, 2023, (N=14)

Item	Mean (Standard Deviation)	I-CVI	CVC	*p* value
Clarity and Relevance of Content				
The language used is easily understandable for the user.	4(0.82)	0.86	0.81	0.02
The information is logically organized.	4 (0.51)	1	0.89	0.79
The information adequately covers the proposed content.	5(0.51)	1	0.91	0.79
The cited references add value to the information.	4(0.77)	0.93	0.83	0.30
The selected topics (Kangaroo Method, Breastfeeding, Sleep and Rest, Hygiene Care, Warning Signs, and Neuropsychomotor Stimulation) are relevant to preterm infant home care.	5(0.42)	1	0.96	0.05
The text on the "Kangaroo Method" topic is relevant to the care of preterm newborns at home.	5(0.64)	0.93	0.91	0.03
The text on the "Breastfeeding" topic is relevant to the care of preterm newborns at home.	5(0.63)	0.93	0.93	0.08
The text on the "Sleep and Rest" topic is relevant to the care of preterm newborns at home.	5(0.63)	0.93	0.93	0.08
The text on the "Hygiene Care" topic is relevant to the care of preterm newborns at home.	5(0.64)	0.93	0.91	0.03
The text on the "Warning Signs" topic is relevant to the care of preterm newborns at home.	5(0.57)	0.93	0.96	0.00
The text on the "Neuropsychomotor Stimulation" topic is relevant to the care of preterm newborns at home.	5(0.65)	0.93	0.90	0.71
Application Appearance				
The graphic design encourages user engagement.	4.6(0.49)	1.00	0.93	0.42
The selected images align with the related texts.	4.5(0.51)	1.00	0.90	1.00
The resources used facilitate access to information.	4.6(0.49)	1.00	0.93	0.42
The application has an attractive visual design.	4.5(0.65)	0.93	0.90	0.71
The application is clearly organized to facilitate topic navigation.	4.6(0.49)	1.00	0.93	0.42
The presentation of the content contributes to the home care of preterm newborns.	4.5(0.65)	0.93	0.90	0.71
Overall Mean I-CVI (S-CVI/AVE) and Overall Mean CVC		0.96	0.91	

* *S-CVI/AVE – Average Content Validity Index across items. (AVE – average variance extracted)*.

Certainly, the content of the items Kangaroo Method, Breastfeeding, Sleep and Rest, Hygiene Care, Warning Signs, and Neuropsychomotor Stimulation was validated by specialists regarding relevance, obtaining an index above 0.80, thereby confirming the validity of the content.

Similarly, the application’s appearance was also validated by the expert panel. The graphic design, the relationship between text and images available for viewing, the resources used for information access, and the facilitated presentation of the content all received an I-CVI and CVC above 0.80.

Regarding initial access to the application, one of the specialists suggested, after analysis, that a login registration section be included rather than only allowing direct login to the screens. Therefore, this suggestion was implemented, and the registration screen was reorganized with the description “Create an Account,” enabling users to create a personal account through the initial screen.

During the validation process, the specialists were also able to provide open-ended comments in the evaluation instrument. Their feedback, including suggestions for improvements and commendations, was analyzed qualitatively, synthesized, and addressed according to the feasibility of modifying the application’s structure and the relevance of the suggestions to the theme.

The suggestions, whether implemented or not, were summarized and listed in Chart 1.

**Chart 1:** Summary of the qualitative analysis of the judges’ suggestions related to the application’s content, Rio de Janeiro, 2023, (N=14)

Comments and Suggestions	Implemented
I suggest that the text use clearer language. At times, technical terms are used, which may create confusion for the user. We do not know the audience that will access it. A population with a lower level of education may have difficulty understanding.	Yes
I suggest adding educational videos after the provided information, as I believe they would facilitate comprehension. Example: a text about bathing… followed by a video on how to do it. A text about changing diapers… followed by a video on how to do it.	No
I suggest removing the references from the text boxes with guidance.	Yes
Some topics could be more illustrated, with less text.	No
I recommend including Tummy Time starting at six months of age.	No
As a suggestion: levels of information (easy, medium, and difficult), where users could answer questions, and depending on their accuracy, they would move up levels or be directed to answers with references.	No
I suggest adding more images to illustrate the correct positioning of the mother and baby during breastfeeding (latch and position) with slides dedicated solely to images. Likewise, images illustrating the bathing process step by step, as seen in the Kangaroo Method handbook from the Ministry of Health.	No
The language is not very informal, and the use of certain terms may create confusion and distance for those using the app (e.g., thermal control, limb flexion, oral cavity, auditory impairment, neonate).	Yes
The automatic transition of slides was somewhat bothersome, as it did not allow enough time to read calmly before moving to the next slide.	Yes
I think it would be helpful to emphasize that milk donation centers also provide assistance to women experiencing breastfeeding difficulties.	Yes
It is also important to better explain some introduced concepts, such as “What is neurostimulation?” in simple and direct terms before describing its benefits and how to stimulate. The same applies to kinesitherapy, which is not clearly defined. Additionally, discussing screen exposure as a negative stimulation factor could be beneficial.	No
For warning signs, it is necessary to explain more clearly what thoracic changes are, using simple explanations for terms like retraction, nasal flaring, cyanosis, and grunting, with illustrations.	Yes
In the hygiene section, consider improving how genital hygiene is described rather than stating it should be done “from inside out.”	Yes
Regarding soap use, instead of just stating that it should not alter pH, specify “neutral soap,” which is easier to understand.	No
Regarding umbilical stump hygiene, there is no mention of the new recommendation advising against using 70% alcohol, instead emphasizing that it should remain dry. It should be explained that using or not using alcohol does not affect the time of stump detachment or infection prevention, and the most crucial factor is keeping it dry. However, alcohol use may be recommended if necessary.	Yes
I suggest minor adjustments to “scientific” words to improve comprehension for non-professional users.	Yes
I believe the psychomotor stimulation section may need reconsideration. Perhaps renaming the tab would make it more understandable for mothers.	No
Increase the time interval between topics, as it is currently too fast. Check if it is possible to allow topic navigation by tapping or swiping the text instead of limiting it to the bottom navigation.	Yes
Avoid using overly technical scientific language. Terms such as “neonatal mortality risk indicator,” “metabolic disorders” (better to specify which ones), and “apathy” (definition) should be clarified.	Yes
The text could contain less information and be formatted into bullet points.	No
I am unsure whether we need to cite references for the family. They should be used for content development but not necessarily cited.	Yes
Some words could be replaced for better comprehension and engagement with the target audience. Despite efforts to use clear language, there are still biomedical terms such as “lateralize” and “ventral” that could be simplified.	Yes

**Figure 1:**
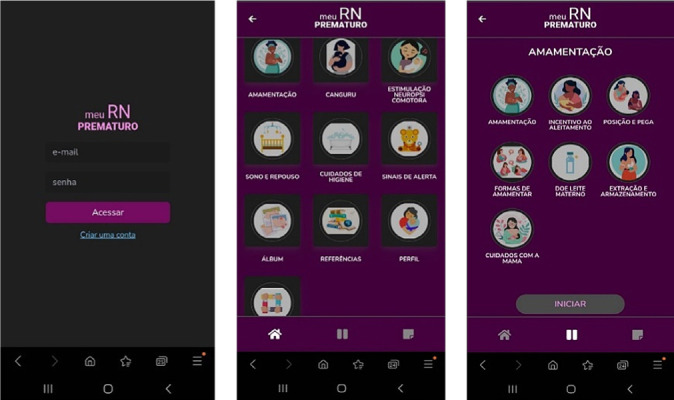
Screenshots of the technological interface of “*Meu RN Prematuro*”

These findings demonstrated that, in just one round of validation conducted by the expert panel, the technological tool was deemed valid. Thus, following the final methodological phase, adjustments related to content and appearance, as well as the organization of data, the arrangement of images on the screens by topic, and compliance with the recommendations highlighted by the expert panel, the application was finalized and defined, as shown in Figure 1.

## DISCUSSION

The developed technological tool provided relevant content for neonatal care, as it was validated by an expert panel in the field. Therefore, considering that most of the Brazilian population has access to mobile networks, digital health can significantly contribute to the continuity of healthcare^(15)^.

Thus, given that one of the criteria for satisfactory validation of the instrument’s content is achieving a CVI and CVC above 0.80^(16)^, it was found that, after the validation round with experts, scores exceeded this threshold. Supporting this finding, a study on the development and content validation of a mobile application on breastfeeding showed approval from the majority of evaluating judges^(17)^.

From this perspective, beyond its use by patients, healthcare professionals can also explore the application, as this technological tool can support decision-making by providing precise and reliable information. Additionally, it facilitates the sharing of topics related to maternal and child health among multidisciplinary teams, thereby enhancing connectivity between professionals and users^(17)^.

Currently, various technological devices designed for the care of preterm newborns outside the hospital setting have demonstrated significant relevance in improving caregiver performance, especially among mothers. These devices also provide rapid access to commonly sought information and enable real-time information sharing^(18)^.

With the advancement of the digital environment, mHealth technologies have emerged as digital health support tools, including applications, websites, and other electronic resources accessible to the population. These technologies can enhance individuals’ understanding of their health through information and communication technology^(15-19)^.

Indeed, applications are popular tools among caregivers, regardless of their level of health literacy. However, there is an ongoing academic debate on whether such easily accessible devices impact child health, given the risk of exposure to inaccurate information^(20)^.

Therefore, the advancement of technology and the various possibilities for health-related applications have led the World Health Organization to establish the Global Observatory for eHealth. This initiative aims to provide highly relevant guidance and support governments in decision-making regarding the use of technology in healthcare^(21,22)^.

Unfortunately, technological connectivity within the SUS remains largely theoretical for the population, as the system is not yet modernized and lacks the technological mechanisms necessary to meet the demands of millions of people, including both Brazilian citizens and immigrants residing in the country who seek this care^(21)^.

Thus, considering Primary Healthcare as the primary point of access to care underscores the positive impact of digital technologies. These technologies serve as valuable tools for healthcare professionals in decision-making and for civil society, integrating applications into public health policies as a means of strengthening collective efforts^(8)^.

From this perspective, considering that approximately 15 million preterm births occur worldwide each year and that, in Brazil, the number of premature births reaches 340,000 annually—equivalent to 931 per day or, more specifically, 6 preterm infants every 10 minutes, according to data from the Brazilian Ministry of Health—developing solutions to enhance neonatal care and prevent hospital readmissions due to avoidable causes is not only necessary but essential^(23)^.

Thus, given the high rates of prematurity in Brazil and globally, and as a means of improving post-discharge care for preterm newborns, the application *Meu RN Prematuro* was created and developed as a user-friendly technological tool made freely available to the public.

Furthermore, conducting field research with the target population of a technology ensures that its content aligns more closely with the reality experienced by caregivers of preterm infants. Therefore, in addition to feedback from the target audience, the application was rigorously based on scientific literature, making it a tool rich in scientific and technological material^(24)^.

Additionally, to become an effective support tool for preterm newborn care and to be widely used by its intended population—as well as to be integrated into public health policies—the application underwent a validation process conducted by an expert panel, which assessed both its content and appearance.

Although a semantic evaluation was not conducted with the application’s target audience, the validation of the “language” item by specialists was essential for modifying and replacing complex scientific terms with more accessible language. This approach aligns with studies asserting that language plays a fundamental role in shaping an individual’s identity and that both written and spoken dialogue are integral to interpersonal interaction^(18,21,25)^.

Since the application’s text was designed for use by mothers of preterm newborns at home, the suggestion to remove bibliographic references at the end of each section was accepted and implemented. This decision was made to ensure that the text remained concise and did not overwhelm or confuse users^(17,21)^.

Another suggestion addressed automatic screen transitions and the time allotted for reading each section. Experts emphasized the need for additional time to review the available material, as the previous duration was too short, preventing users from fully engaging with the content. Moreover, to complete the reading, users often had to navigate back to previous screens multiple times.

Undeniably, evaluating the user experience in the use of an application ensures that specific adjustments enhance usability^(6,8,25)^. Thus, in the present study, comments related to screen time were considered, ensuring longer viewing time for information as well as the possibility of switching topics—an option that already existed based on user preferences.

According to national and international literature, integrating the family into the care of preterm newborns during hospitalization provides benefits related to neuropsychomotor development. Specifically, skin-to-skin mother-infant contact accelerates brain maturation, while breastfeeding contributes to cognitive development in preterm infants^(20,26)^.

Thus, despite expert evaluations questioning the need to maintain the neuropsychomotor stimulation topic, no modifications were made to the application, and all content was preserved and justified based on the available literature.

Additionally, the content on neuropsychomotor stimulation was validated by the expert panel, reinforcing its relevance for the target audience at home and its potential benefits for preterm infants, such as reducing neonatal health risks associated with prematurity.

Furthermore, user interaction with the application, including the ability to input personal information, was designed to engage users while also providing valuable material for healthcare professionals in future consultations, particularly during follow-up care in pediatric health assessments^(27)^.

The *Meu RN Prematuro* application was primarily designed as a maternal support tool outside the tertiary healthcare network, serving as a resource to be accessed after hospital discharge. It enables mothers to make informed decisions regarding neonatal care and enhances their confidence in home-based care.

However, after analyzing expert responses, it became evident that this tool extends far beyond merely providing content to mothers of preterm newborns. It is also a valuable health education resource for public health, given its high level of relevance in neonatal care.

Moreover, the ability to access scientific materials, either physically or remotely, can contribute to resolving health-related concerns for preterm infants across different regions of the country. This ensures user-centered care tailored to individual needs, ultimately preventing adverse health outcomes.

### Study limitations

The present study has certain limitations, including the small sample size of mothers, which may not be representative of the total population of mothers of preterm infants. Additionally, semantic evaluation was not included in this research phase. Moreover, the involvement of other professionals responsible for neonatal care could have broadened the study’s scope. However, the findings may encourage researchers to further evaluate this application and investigate its practical utility in the daily lives of mothers of preterm newborns.

### Contributions to Nursing and Healthcare

The development of a technology based on scientific literature and evaluated by expert nurses in the field, aimed at preterm infant care, is not only innovative but also essential, given the ongoing concerns surrounding premature births. Furthermore, maintaining continuity of care outside the hospital network and strengthening the interconnection between healthcare services is of utmost importance to the Brazilian population. This can potentially be achieved through the use of this technological tool.

## CONCLUSIONS

The study successfully achieved its proposed objective and demonstrated, through expert evaluation, adequate content validation, confirming that the developed technology is valid, easy to use, publicly beneficial, innovative, and scientifically relevant and accessible.

Thus, in addition to its usefulness for mothers and families of preterm newborns, this technological tool also serves as a valuable resource for nurses, as it enhances initiatives aimed at promoting and protecting child health by providing targeted care and clear guidance for caregivers.

Furthermore, as a recommendation, future studies should focus on the semantic and cross-cultural validation of this mobile application to support mothers of preterm newborns, with the goal of making this tool applicable in international settings.

## Data Availability

Not applicable.
