# Supplementary material for: Construction and validation of a mobile technology to support mothers of preterm newborns
Source: Rev Bras Enferm. 2025 Sep 1;78(Suppl 4):e20240336. doi: 10.1590/0034-7167-2024-0336 (PMC12419780; doi:10.1590/0034-7167-2024-0336)
Supplement: Translation

# Translation: Construção e validação de uma tecnologia móvel para apoio às mães de recém-nascidos prematuros

**DOI:** 10.1590/0034-7167-2024-0336pt

**Authors:** Thaís Emanuele da Conceição, Maria Helena do Nascimento Souza, Rafael Braga Esteves, Patrícia Lima Pereira Peres, Donatella Valente, Giovanni Galeoto, Antonella Nespoli

**Affiliations:** I Universidade Federal do Rio de Janeiro. Rio de Janeiro, Rio de Janeiro, Brazil; II Universidade Estadual do Rio de Janeiro. Rio de Janeiro, Rio de Janeiro, Brazil; III Sapienza Università di Roma. Lácio, Roma, Itália; IV Universidade de Milano-Bicocca. Milão, Lombardia, Itália

**Keywords:** Aplicativos Móveis, Recém-Nascido Prematuro, Alta do Paciente, Assistência Domiciliar, Cuidados de Enfermagem

## Abstract

**Objetivos:**

construir e validar o conteúdo de uma tecnologia móvel como ferramenta tecnológica para o cuidado ao recém-nascido pré-termo no domicílio.

**Métodos:**

estudo metodológico de caráter descritivo, qualitativo e quantitativo, que consistiu na elaboração de um aplicativo, construído e validado a partir de três etapas: levantamento bibliográfico e análise das entrevistas com as mães; elaboração do aplicativo; e validação de conteúdo por painel de especialistas.

**Resultados:**

por meio da revisão integrativa da literatura e análise das entrevistas com 10 mães, foi possível identificar os temas inseridos na tecnologia, que foram validados por 14 especialistas, com índice e coeficiente de validade de conteúdo superiores a 0,80 nos 17 itens avaliados e consistência interna de α=92,6.

**Conclusões:**

a construção do aplicativo para mães de recém-nascidos prematuros revelou elevado grau de concordância pelo painel de especialistas, demonstrando que o conteúdo desenvolvido é válido e constitui mais uma tecnologia de cuidado para o enfermeiro.

## INTRODUÇÃO

A longa caminhada que o Brasil percorreu desde a década de 1980 demonstrou que houve inúmeros avanços científicos e tecnológicos na saúde neonatal, influenciando uma expressiva redução da mortalidade infantil e neonatal. No entanto, a prematuridade, definida como o nascimento antes de 37 semanas de idade gestacional, ainda é um problema de saúde pública mundial^(1)^.

Apesar de evitável, calcula-se que, aproximadamente, 12% dos nascimentos que ocorrem em território nacional sejam prematuros, um índice elevado em comparação aos países desenvolvidos^(1)^. Além disso, atualmente, a taxa de mortalidade neonatal no Brasil é de 7,87, estando acima da meta nacional estabelecida nos Objetivos de Desenvolvimento Sustentável (ODS) para 2030, que preconiza menos de 5,0 óbitos neonatais por mil nascidos vivos, a fim de reduzir as vulnerabilidades populacionais^(2)^.

De fato, as crianças nascidas prematuras são pacientes que necessitam de assistência integral. Nessa perspectiva, ressalta-se a importância do acompanhamento dos profissionais da rede de atenção primária à saúde, especificamente daqueles vinculados à equipe de saúde da família, que devem coordenar o cuidado integral prestado às famílias residentes no território de abrangência da sua unidade, assim como dispensar orientações acerca dos cuidados diários com o bebê^(3,4)^.

Ademais, considerando o cenário brasileiro e suas particularidades relacionadas à saúde, a Atenção Primária à Saúde enquadra-se como uma estratégia de atenção integral à saúde da população neonatal e de suas famílias, diante dos desafios para a materialização dos ODS e da execução da Agenda 2030. O princípio de “Não deixar ninguém para trás” representa um importante desafio para alcançar esse atendimento integral, independentemente da condição social da população^(2)^.

Evidentemente, a experiência do parto prematuro para as famílias pode romper expectativas construídas ao longo da gestação, em razão da hospitalização do filho, do momento da alta e da transição para o domicílio, podendo estar envolta em sofrimentos e dificuldades^(5)^. Assim, para além da assistência prestada nos diversos níveis da rede de atenção à saúde, o uso de tecnologias, como aplicativos, contribui para a eficácia da educação em saúde e representa um importante suporte para a continuidade da assistência, pois auxilia no acesso às orientações fornecidas desde a internação hospitalar até a alta para o domicílio, favorecendo um cuidado mediado pela tecnologia^(6)^.

Outrossim, o uso da tecnologia tem se tornado um grande aliado no que tange à disseminação de informação correta, permitindo que a assistência dos profissionais, em especial da enfermagem, esteja atrelada ao uso de ferramentas validadas, que sejam disponibilizadas para a clientela sempre que necessário. Isso possibilita, assim, que usuários do Sistema Único de Saúde (SUS) criem proximidade com o ambiente tecnológico^(6,7)^.

Assim, diante da magnitude dessa problemática e das evidências de insuficiência de aplicações relacionadas à enfermagem neonatal existentes no meio digital com vistas à vinculação em programas de saúde pública, este estudo ressalta a relevância das investigações relacionadas ao uso da tecnologia no cuidado ao recém-nascido pré-termo no contexto domiciliar e destaca a necessidade da realização de estudos que avaliem a pertinência e a adequação de uma nova ferramenta tecnológica^(8)^.

## OBJETIVOS

Construir e validar o conteúdo de uma tecnologia móvel como ferramenta tecnológica para o cuidado ao recém-nascido pré-termo no domicílio.

## MÉTODOS

### Aspectos éticos

O presente estudo atendeu à Resolução nº 466/12, à Carta Circular nº 1 de 03 de março de 2021 e foi aprovado em 13 de abril de 2022 pelo Comitê de Ética em Pesquisa da Universidade Federal do Estado do Rio de Janeiro – Escola de Enfermagem Anna Nery. Aos participantes foi solicitada a concordância com o Termo de Consentimento Livre e Esclarecido (TCLE), enviado por meio eletrônico, através da plataforma Formulários Google e/ou assinado de forma presencial.

### Tipo de estudo

Estudo metodológico de caráter descritivo, que consistiu na construção e validação de conteúdo de um aplicativo a ser utilizado no cuidado ao bebê pré-termo no ambiente domiciliar. O estudo foi realizado com base na ferramenta *Consolidated Criteria for Reporting Qualitative Research* (COREQ), conforme preconiza a Rede EQUATOR, que orienta a descrição de estudos qualitativos^(9)^.

### Cenário do estudo

O cenário do estudo foi uma Unidade de Terapia Intensiva Neonatal (UTIN) de uma maternidade especializada no atendimento à gestante de risco, situada no município do Rio de Janeiro.

### População e critérios de seleção

Para a primeira etapa do estudo, referente à definição do conteúdo teórico a ser incorporado no aplicativo, a pesquisadora contou com dez participantes, mães de recém-nascidos pré-termo, selecionadas a partir de uma amostra de conveniência, tendo como critério de inclusão: ser mãe de recém-nascido pré-termo que nasceu e foi internado na UTIN. Foram excluídas as mães com idade inferior a dezoito anos e mães de recém-nascidos que apresentaram malformações congênitas ou que, após a alta hospitalar, necessitavam de dispositivos invasivos e cuidados domiciliares especializados.

Após a segunda etapa, referente à elaboração do protótipo do aplicativo, foram selecionados os participantes para efetuarem a validação do aplicativo. Nesta terceira etapa, foram selecionados quatorze enfermeiros considerados avaliadores especialistas.

A identificação dos especialistas ocorreu por meio de uma busca na Plataforma Lattes do site do Conselho Nacional de Desenvolvimento Científico e Tecnológico (CNPq) e pela técnica de amostragem não probabilística *snowball*, que consiste na indicação de novos participantes pelos primeiros^(7)^.

Como critério de inclusão dos enfermeiros especialistas, foram adotados os requisitos estabelecidos por Jasper (1994)^(10)^: possuir habilidade/conhecimento adquiridos pela experiência; possuir habilidade/conhecimento que torne o profissional uma autoridade no assunto; ou possuir aprovação em um teste específico para identificar especialistas. Nesta investigação, estipulou-se que os profissionais deveriam atender, minimamente, a dois desses requisitos para serem identificados como especialistas na área temática do aplicativo elaborado.

Dessa forma, foram identificados 27 (vinte e sete) especialistas, todos enfermeiros com especialização e experiência profissional na área de neonatologia e conhecimento acerca dos cuidados com o recém-nascido pré-termo. Destes, 14 (quatorze) responderam à pesquisa, mediante questionário eletrônico enviado por e-mail fornecido após contato telefônico, sendo a amostra adequada no que tange aos estudos de validação de conteúdo^(11)^.

### Protocolo do estudo

O estudo foi desenvolvido em três etapas: levantamento bibliográfico e análise das entrevistas com as mães; elaboração do aplicativo e; validade de conteúdo por painel de especialistas, respeitando a metodologia estabelecida pelo modelo Design de Interação Centrado no Usuário, sendo um referencial de estruturação que estipula a participação do destinatário final na etapa de criação de sistemas informatizados, como os aplicativos móveis^(12)^.

Desta forma, as etapas do estudo foram:

### 1ª Etapa - Levantamento bibliográfico e análise das entrevistas com as mães

Para a primeira etapa, foi realizada uma revisão de literatura em seis bases de dados: *Exerpta Medica Database* (EMBASE), *Medical Literature Analysis and Retrieval System Online* (Medline) via PubMed, *Web of Science* via Portal de Periódicos da CAPES, *Literatura Latino-Americana em Ciências da Saúde* (LILACS), *Scientific Electronic Library Online* (SciELO) e *Cochrane Library*. As buscas foram realizadas nas bases de dados eletrônicas entre os meses de janeiro e maio de 2022, tendo como pergunta norteadora: “Quais são as produções científicas publicadas no período de 2012 a 2021 acerca das dúvidas maternas no cuidado com o recém-nascido prematuro no domicílio?”.

As estratégias de busca incluíram descritores controlados, pesquisados no *Descritores em Ciências da Saúde* (DeCS) e *Medical Subject Heading* (MeSH) via PubMed, no idioma inglês e seus sinônimos, além de descritores não controlados, todos conectados pelos operadores booleanos *AND* e *OR*, além do uso de símbolos, como o de truncamento, a depender das características de cada base pesquisada.

Ainda nesta primeira etapa do estudo, foram realizadas entrevistas semiestruturadas com as seguintes perguntas abertas: “Quais as suas dúvidas com relação aos cuidados com seu filho?”; “De todos os cuidados com o recém-nascido pré-termo, o que mais te traz medo em fazer sozinha?”; “Que informações você gostaria de ter à disposição quando estiver no domicílio?”; e “Quem são as pessoas que compõem a sua rede de apoio?”.

As entrevistas com as mães ocorreram entre os meses de agosto e setembro de 2022, em um espaço reservado dentro da unidade hospitalar onde os recém-nascidos eram acompanhados após a alta da UTIN, e tiveram duração média de dez minutos. Participaram da pesquisa dez mães de neonatos pré-termo, cuja idade variou de 20 a 44 anos, que ainda estavam internados na UTIN, com o objetivo de complementar a pesquisa e elencar, de forma prática, os desafios enfrentados pelas cuidadoras.

Assim, após a revisão de literatura, que elencou 21 artigos publicados em periódicos, classificados em sua maioria com nível de evidência VI, e após a realização das entrevistas maternas, finalizadas após a saturação dos conteúdos relatados, transcritas e analisadas na íntegra, evidenciou-se que as principais dúvidas estavam relacionadas à amamentação, cuidados de higiene, cólica, banhos de sol, identificação de sinais de alerta e ao método canguru. Dessa forma, foram elencados seis grandes temas que subsidiaram a construção do aplicativo para o cuidado ao recém-nascido pré-termo no domicílio: Canguru; Amamentação; Sono e repouso; Cuidados de higiene; Sinais de alerta; e Estimulação neuropsicomotora.

### 2ª Etapa – Elaboração do aplicativo

Com base nos assuntos elencados para a concepção e construção teórica da tecnologia, foi idealizado um modelo de aplicativo móvel, desenvolvido online por meio do site gratuito *Canva.com*, com imagens de domínio público projetadas com base nas etapas anteriores e seguindo o modelo metodológico estabelecido.

Na idealização do material, a tela inicial, após a tela de capa, foi composta por um logotipo constituído por pés que representam os de recém-nascidos e pelo nome *Meu RN Prematuro*, na cor roxa, por ser utilizada para simbolizar o Dia Mundial da Prematuridade^(13)^.

A tela seguinte foi composta por campos para inserção de dados de identificação, como nome do bebê, data de nascimento, idade gestacional corrigida, peso e data da alta hospitalar, cujo preenchimento será realizado pela mãe ou pelo profissional enfermeiro da unidade no momento em que o binômio receber alta para o domicílio.

O item “idade gestacional corrigida” foi inserido em virtude do impacto da prematuridade no crescimento e desenvolvimento do neonato. Dessa forma, esse item visa contribuir para a continuidade do cuidado na rede de atenção primária à saúde, tendo em vista que, para o recém-nascido pré-termo, há gráficos de avaliação próprios desenvolvidos para utilização nas consultas de seguimento.

A tela seguinte foi elaborada com o intuito de promover o vínculo entre o neonato e sua família, possibilitando a inserção de recortes fotográficos realizados durante o período de internação, de acordo com a vontade da cuidadora e demais envolvidos.

### 3ª Etapa - Validação de conteúdo por painel de especialistas

Foram 14 (quatorze) participantes que responderam à pesquisa com vistas à validação do conteúdo do aplicativo, sendo todas do sexo feminino, com idade variando entre 25 e 60 anos, sendo que o maior percentual encontrava-se na faixa etária de 36 a 45 anos (64,3%).

Os membros do painel de especialistas residiam e atuavam profissionalmente no estado do Rio de Janeiro/RJ, na rede pública de saúde, possuindo ampla experiência profissional em hospitais com atendimento especializado ao neonato pré-termo. Destes, 92,9% possuíam cinco anos ou mais de exercício profissional.

Para a análise dos resultados, foi elaborado um banco de dados no programa *Excel*, onde cada questão do instrumento estruturado para a validação do aplicativo, no que se refere à relevância do conteúdo, linguagem escrita, atratividade para o usuário e pertinência dos temas, recebeu um código numérico com base nas cinco possibilidades de resposta: “discordo fortemente (1)”, “discordo (2)”, “não concordo nem discordo (3)”, “concordo (4)” e “concordo fortemente (5)”.

Para a análise de validade e confiabilidade dos itens contidos no aplicativo, os especialistas avaliaram os critérios utilizando o software *Statistical Package for the Social Sciences* (SPSS), com o cálculo do Índice de Validade de Conteúdo (IVC) e do Coeficiente de Validade de Conteúdo (CVC) de cada item. O critério de validação adotado foi um valor superior a 0,80^(11)^, considerando a média das respostas “4” e “5” atribuídas pelo painel de especialistas.

Para verificar a consistência interna, foi calculado o alfa de Cronbach, sendo que valores entre 0,70 e 0,95 indicam forte correlação. Além disso, foi realizado o teste exato de distribuição binomial, adotando como critério de significância p>0,05.

## RESULTADOS

Os resultados da primeira etapa subsidiaram a construção do conteúdo do aplicativo.

A partir da revisão sistemática da literatura, na qual foram analisados 21 artigos publicados no recorte temporal de 2012 a 2021, identificou-se que as principais dúvidas das mães frente ao cuidado ao recém-nascido prematuro no domicílio estão relacionadas à amamentação, higiene, banho de sol, cólica da criança, alterações clínicas, temperatura e método canguru^(14)^.

Nesta etapa, considerou-se ainda o resultado das entrevistas realizadas com as dez mães de crianças nascidas entre a 25ª e a 36ª semana de gestação. Essas mães tinham idade entre 20 e 44 anos; a maioria era solteira (6), possuía ensino fundamental (6) e não tinha trabalho remunerado fora do lar (6).

Com relação às incertezas relacionadas ao cuidado do prematuro, as mães referiram aspectos ligados ao aleitamento materno, cuidados de higiene, oferta de leite artificial, identificação de sinais de anormalidades, sono e repouso, como ilustrado nos seguintes trechos:

*Minha única dúvida, em casa, eu acho que vai ser sobre alimentação. Porque o estômago dela é menor, qual será o tempo certo para amamentar.* (Mãe ٠٦)
*Será que eu vou conseguir dar banho nessa criança?* [...] *Mas eu queria ter mais confiança na hora do banho.* (Mãe 03)
*Eu estou vendo que ela está tendo uma dificuldade em sugar a mamadeira, vejo que ela demora muito.* (Mãe 08)
*Ele precisou usar oxigênio, então, em casa, eu vou ter que prestar mais atenção nisso. Se ele passar mal, não sei se saberia identificar, acho que não.* (Mãe 05)
*A minha preocupação com ele é sobre a respiração em casa.* [...] *A respiração me preocupa muito. E o vômito, isso dele colocar tudo para fora.* (Mãe 09)
*Nunca fui mãe. Tenho dúvidas com relação ao banho, com a forma de colocar para dormir.* (Mãe 10)

Com os resultados da revisão da literatura e os dados obtidos nas entrevistas, foi possível estruturar o conteúdo do aplicativo e prosseguir para a validação dos especialistas.

Assim, com relação à estrutura e disponibilização dos temas do aplicativo, foi realizada uma organização a fim de dar sentido ao conteúdo, ou seja, a sequência de informações disponíveis na tela principal possibilitará ao usuário a conexão entre os temas, proporcionando, dessa forma, uma organização temática acerca do cuidado.

Além disso, todo o conteúdo foi estruturado respeitando as regras da Língua Portuguesa em vigor no território nacional e revisado por um especialista na temática antes de ser inserido e disponibilizado no aplicativo, proporcionando, assim, coerência, adequada concepção da linguagem e atendimento às regras gramaticais. Para tanto, a avaliação semântica não foi contemplada nesta etapa do estudo.

Com relação ao perfil dos participantes do painel de especialistas, verificou-se que estes possuíam especialização na área de neonatologia (42,9%), seguida por mestrado (28,6%) e doutorado (28,6%).

Os dados relacionados à caracterização do painel de especialistas estão descritos na Tabela 1.

**Tabela 1:** Perfil do painel de especialistas, Rio de Janeiro, 2023, (N=14)

Características	n	%
Idade (em anos)		
25 – 35	03	21,4
36 – 45	09	64,3
46 – 60	02	14,3
Formação Acadêmica		
Especialização	06	42,9
Mestrado	04	28,6
Doutorado	04	28,6
Tempo de Experiência Profissional na área de Enfermagem em Neonatologia (em anos)		
3 a 4	01	7,1
5 ou mais	13	92,9

Em relação aos itens avaliados pelos especialistas, notou-se que o I-IVC médio foi de 0,96 e o Coeficiente de Validade de Conteúdo (CVC) médio foi de 0,91 (Tabela 2), refletindo excelentes avaliações de concordância no que diz respeito ao conteúdo e à aparência da versão final do aplicativo proposto, que apresentou uma consistência interna de α = 92,6.

**Tabela 2:** Avaliação dos especialistas com relação à clareza, relevância e à aparência do aplicativo, de acordo com o cálculo do Índice de Validade de Conteúdo e do Coeficiente de Validade de Conteúdo, Rio de Janeiro, 2023, (N=14)

Item	Média (Desvio Padrão)	I-IVC	CVC	Valor de *p*
Clareza e Relevância do conteúdo				
A linguagem utilizada é de fácil compreensão pelo usuário.	4(0,82)	0,86	0,81	0,02
As informações estão organizadas de forma lógica.	4 (0,51)	1	0,89	0,79
As informações abrangem adequadamente o conteúdo proposto.	5(0,51)	1	0,91	0,79
As referências citadas acrescentam valor à informação.	4(0,77)	0,93	0,83	0,30
Os temas elencados (Canguru, Amamentação, Sono e repouso, Cuidados de higiene, Sinais de alerta e Estimulação neuropsicomotora) são pertinentes ao cuidado com o prematuro no domicílio.	5(0,42)	1	0,96	0,05
O texto relacionado ao tema “Canguru” possui relevância no cuidado com o recém-nascido prematuro no domicílio.	5(0,64)	0,93	0,91	0,03
O texto relacionado ao tema “Amamentação” possui relevância no cuidado com o recém-nascido prematuro no domicílio.	5(0,63)	0,93	0,93	0,08
O texto relacionado ao tema “Sono e repouso” possui relevância no cuidado com o recém-nascido prematuro no domicílio.	5(0,63)	0,93	0,93	0,08
O texto relacionado ao tema “Cuidados de higiene” possui relevância no cuidado com o recém-nascido prematuro no domicílio.	5(0,64)	0,93	0,91	0,03
O texto relacionado ao tema “Sinais de alerta” possui relevância no cuidado com o recém-nascido prematuro no domicílio.	5(0,57)	0,93	0,96	0,00
O texto relacionado ao tema “Estimulação neuropsicomotora” possui relevância no cuidado com o recém-nascido prematuro no domicílio.	5(0,65)	0,93	0,90	0,71
Aparência do App				
O design gráfico estimula a utilização pelo usuário.	4,6(0,49)	1,00	0,93	0,42
As imagens selecionadas condizem com os textos relacionados.	4,5(0,51)	1,00	0,90	1,00
Os recursos utilizados proporcionam o acesso às informações.	4,6(0,49)	1,00	0,93	0,42
O aplicativo possui visual atraente.	4,5(0,65)	0,93	0,90	0,71
O aplicativo está organizado de forma clara para facilitar a localização dos assuntos.	4,6(0,49)	1,00	0,93	0,42
A forma de apresentação dos conteúdos contribui para o cuidado do recém-nascido prematuro no domicílio.	4,5(0,65)	0,93	0,90	0,71
IVC médio geral (S-IVC/AVE) e CVC médio geral		0,96	0,91	

* *S-IVC/AVE – Índice de validade de conteúdo médio entre os itens; (AVE – average variance extracted)*.

Decerto, os conteúdos dos itens Canguru, Amamentação, Sono e repouso, Cuidados de higiene, Sinais de alerta e Estimulação neuropsicomotora foram validados pelos especialistas quanto à relevância e obtiveram um índice acima de 0,80, tornando o conteúdo válido.

De igual forma, a aparência do aplicativo também foi validada pelo painel de especialistas, no qual o design gráfico, a relação entre texto e imagem disponível para visualização, os recursos utilizados para o acesso à informação e a apresentação facilitada do conteúdo também obtiveram um I-IVC e um CVC acima de 0,80.

Já com relação ao acesso inicial ao aplicativo, um dos especialistas sugeriu, após análise, que houvesse um local para a realização de cadastro de login e não apenas o login com acesso direto às telas. Portanto, a sugestão foi atendida, e foi realizada uma reorganização da tela de cadastro com a descrição “criar uma conta”, sendo possível, por meio da tela inicial, a criação de um cadastro pessoal.

Ainda no processo de validação, os especialistas puderam registrar, no instrumento de avaliação, comentários livres, cujas considerações relacionadas a melhorias e elogios foram analisadas qualitativamente, sintetizadas e atendidas de acordo com a possibilidade de alterações na estrutura do aplicativo e a pertinência ao tema.

As sugestões atendidas ou não foram sintetizadas e elencadas no Quadro 1.

**Quadro 1:** Síntese da análise qualitativa das sugestões dos juízes para o aplicativo relacionada ao conteúdo, Rio de Janeiro, 2023, (N=14)

Comentários e sugestões	Atendido
Sugiro que o texto tenha uma linguagem um pouco mais clara. Em alguns momentos são usados termos técnicos que podem gerar dúvida no usuário. Não sabemos o público que vai acessar. Uma população com menor grau de instrução pode ter dificuldade de compreensão.	Sim
Sugiro que após as informações dadas sejam expostos vídeos educativos, acredito que facilitaria a compreensão do que está sendo explícito. Exemplo: texto sobre o banho... vídeo de como realizar. Texto sobre troca de fraldas... vídeo de como realizar.	Não
Eu sugiro retirar as referências do texto que estão nas caixinhas de orientação.	Sim
Alguns temas poderiam ser mais ilustrados, com menos texto.	Não
Recomendo inserir o Tummy Time a partir dos 6 meses de vida.	Não
Como sugestão: Níveis de informação (leve, médio e difícil) onde talvez elas respondessem a perguntas e conforme os acertos, mudem de nível ou fosse direcionadas as respostas com referência.	Não
Sugiro mais imagens para ilustrar a forma adequada da mãe e bebê durante amamentação (pega e posição) slides apenas para imagens. Assim como também imagens para ilustrar o momento do banho, o passo a passo que tem no caderno do Canguru do MS.	Não
A linguagem não está muito informal, o uso de alguns termos pode gerar incompreensão e distância de quem está usando o app (exemplo: controle térmico, flexão dos membros, cavidade oral, acometimento auditivo, neonato).	Sim
A passagem automática dos slides me incomodou um pouco, pois não dava tempo suficiente para ler com calma sem que o mesmo passasse para o slide seguinte.	Sim
Acho que seria interessante salientar que os locais indicados para doação de leite também são para atendimento a mulheres com dificuldades na amamentação.	Sim
Considero também importante explicar melhor alguns conceitos introduzidos como “o que é a neuroestimulação?” Com palavras simples e direta, para então dizer os benefícios e como estimular. O mesmo em relação à cinesiterapia, não fica claro sobre o que é. Poderia também falar sobre o uso de telas como estimulação negativa.	Não
Nos sinais de alerta, necessário explicar melhor o que são alterações de tórax, explicar de forma simples o que é uma retração, batimento de asa de nariz, cianose, sigilo, usando imagens para ilustrar.	Sim
Na parte de higiene acho que pode melhorar a forma de falar sobre a higiene da genitália ao invés de colocar que deve ser “de dentro pra fora”.	Sim
Sobre uso do sabonete, não só colocar sabonete que não altere o pH, mas dizer sabonete neutro que é mais fácil de compreender.	Não
Sobre higiene do coto, senti falta da explicação sobre ser uma nova recomendação, a não necessidade do uso de álcool a 70%, mas manter seco. Explicar que o uso ou não do álcool não altera o tempo de queda e nem previne mais infecção, que o mais importante é estar seco, porém o uso pode ser indicado caso necessário.	Sim
Sugiro pequenos ajustes nas palavras “científicas” a fim de melhor entendimento dos usuários não profissionais.	Sim
Acredito que no espaço estimulação psicomotora, possa ser necessário rever sua utilidade. Não sei se talvez alterasse o nome da aba, para que as mães entendessem mais fácil.	Não
Aumentar o tempo de mudança entre os temas porque está um pouco rápido, ver se é possível colocar a mudança de tema tocando ou deslizando o texto em vez de ser só na parte inferior.	Sim
Evitar usar a linguagem científica mais elaborada. Termos como “indicador de risco de mortalidade neonatal”, distúrbios do metabolismo - melhor descrever quais são, apatia (definição).	Sim
Texto pode ter menos informações, usar em tópicos.	Não
Tenho dúvida se para a família precisamos citar as referências . Usar para construção do conteúdo, mas não citar.	Sim
Algumas palavras poderiam ser trocadas para melhor compreensão e aproximação do público-alvo; apesar do esforço em usar uma linguagem clara ainda há termos biomédicos como “lateralizar”, “ventral”, entre outros.	Sim

Tais achados evidenciaram que, em apenas uma rodada de validação realizada pelo painel de especialistas, a ferramenta tecnológica foi considerada válida. Assim, após a última etapa metodológica, os ajustes relacionados ao conteúdo e à aparência, além da organização dos dados, das imagens dispostas nas telas por assunto e do atendimento às recomendações salientadas pelo painel de especialistas, o aplicativo foi finalizado e definido, conforme demonstrado através das Figura 1.

**Figura 1:** Capturas de telas da interface tecnológica do “Meu RN prematuro”

## DISCUSSÃO

A ferramenta tecnológica desenvolvida apresentou conteúdo relevante para o cuidado neonatal, uma vez que foi validada por um painel de especialistas na temática. Portanto, considerando que a maior parte da população brasileira possui acesso à rede móvel, a saúde digital pode contribuir significativamente para a continuidade do cuidado à saúde^(15)^.

Assim, levando em consideração que um dos critérios para uma validação satisfatória do conteúdo do instrumento é a observação de IVC e CVC acima de 0,80^(16)^, verificou-se que, após a rodada de validação com os especialistas, obteve-se pontuações superiores a esse valor. Corroborando esse achado, um estudo de desenvolvimento e validação de conteúdo de um aplicativo móvel sobre aleitamento materno mostrou a aprovação da maioria dos juízes avaliadores^(17)^.

Nessa perspectiva, para além da utilização da tecnologia pelo usuário, os profissionais de saúde também podem explorar o aplicativo, uma vez que tal aparato tecnológico pode apoiar a tomada de decisão por meio de informações precisas e confiáveis, além de possibilitar o compartilhamento de tópicos voltados para a saúde materno-infantil entre equipes multidisciplinares, aumentando, assim, a conectividade entre profissional e usuário^(17)^.

Atualmente, diversos dispositivos tecnológicos direcionados ao cuidado do recém-nascido pré-termo fora do ambiente hospitalar têm mostrado significativa relevância no desempenho dos cuidadores, principalmente as mães, além de serem meios de acesso rápido a conteúdos de interesse comum e compartilhamento de informações em tempo real^(18)^.

Com o avanço do meio digital, surgiram as tecnologias de *mHealth*, que são ferramentas de apoio à saúde em formato digital, como aplicativos, websites e demais dispositivos disponíveis para acesso da população por meio eletrônico e que podem favorecer a compreensão acerca da própria saúde por meio da tecnologia da informação e comunicação^(15-19)^.

De fato, as aplicações são ferramentas populares entre os cuidadores, independentemente do grau de instrução em saúde que possuam. No entanto, existe uma discussão no meio acadêmico sobre se tais dispositivos acessíveis afetam ou não a saúde infantil, uma vez que pode haver acesso a informações inverídicas^(20)^.

Portanto, o avanço tecnológico e as diversas possibilidades de aplicações voltadas para a área da saúde fizeram com que a Organização Mundial da Saúde criasse o *Global Observatory for eHealth*, com o intuito de fornecer orientações de alta relevância e apoiar o governo nas tomadas de decisão a respeito da utilização do meio tecnológico na assistência à saúde^(21,22)^.

Infelizmente, a conexão tecnológica no SUS ainda permanece no imaginário da população, já que, atualmente, tal sistema não é modernizado nem possui mecanismos tecnológicos capazes de suprir a demanda dos milhões de seres humanos, incluindo brasileiros e imigrantes, que residem no território brasileiro e buscam esse atendimento^(21)^.

Assim, pensar na Atenção Primária como linha de chegada ao atendimento faz com que a utilização de tecnologias digitais tenha impacto positivo e sirva como ferramenta adequada para os profissionais de saúde nas tomadas de decisão e para a sociedade civil, atrelando os aplicativos às políticas públicas em saúde como uma forma de unir forças^(8)^.

Nesta perspectiva, levando em conta que anualmente nascem cerca de 15 milhões de pré-termos no mundo e que, no Brasil, o número de nascimentos antecipados é de 340 mil por ano, sendo 931 por dia ou, mais especificamente, 6 bebês pré-termo a cada 10 minutos, de acordo com dados do Ministério da Saúde (MS), desenvolver algo que melhore a assistência neonatal e previna novas internações por causas evitáveis não só é necessário, mas indispensável^(23)^.

Então, levando em consideração os altos índices de prematuridade no Brasil e no mundo e como uma forma de melhorar a assistência ao recém-nascido pré-termo visando o pós-alta hospitalar, o aplicativo intitulado *Meu RN Prematuro* foi criado e desenvolvido para ser uma ferramenta tecnológica de fácil acesso e disponibilizada para a população de forma gratuita.

Além disso, o uso de pesquisa de campo com a população-alvo de uma tecnologia permite que seu conteúdo esteja mais próximo da realidade vivida pelos cuidadores de crianças prematuras. Portanto, além das pontuações do público-alvo, a elaboração do aplicativo foi criteriosamente embasada na literatura, tornando a ferramenta rica em material científico e tecnológico^(24)^.

Em adição, para que possa tornar-se um instrumento auxiliar no cuidado ao pré-termo e de uso da população à qual se destina, bem como uma ferramenta vinculada às políticas públicas em saúde, foi realizado o processo de validação por um painel de especialistas, cuja avaliação foi relacionada ao conteúdo e à aparência.

Apesar de a avaliação semântica não ter sido realizada com o público-alvo do aplicativo criado, a validação do item “linguagem” pelos especialistas foi essencial para a modificação e substituição dos termos científicos considerados complexos por palavras de cunho popular, corroborando estudos que afirmam que, por meio da linguagem, ocorre a constituição do sujeito e que o diálogo, escrito ou falado, faz parte dos processos de interação entre os indivíduos^(18,21,25)^.

Tendo em vista que toda a parte textual do aplicativo foi construída para o manuseio das mães de pré-termo no domicílio, as sugestões de retirada das referências bibliográficas ao final de todo o conteúdo foram aceitas e implementadas, uma vez que o cuidado com a densidade das informações textuais é importante para não confundir o destinatário e causar estranheza^(17,21)^.

Outro ponto de sugestão foi a respeito da mudança automática de tela e do tempo disponibilizado para tal, em que a fala dos especialistas salientou a necessidade de mais tempo para leitura do material disponível em cada item, uma vez que o prazo estava sendo curto e sem possibilidade de apreciação de todo o conteúdo em tempo hábil. Além disso, para que fosse realizada toda a leitura, era necessário retornar à tela diversas vezes.

Inegavelmente, a avaliação da experiência do usuário frente à utilização de um aplicativo faz com que certos ajustes se tornem eficazes para a usabilidade^(6,8,25)^. Dessa forma, no presente estudo, os comentários relacionados ao tempo de tela foram acatados, garantindo mais tempo de visualização da informação, além da possibilidade de mudança de assunto, algo que já existia conforme o interesse do usuário.

De acordo com a literatura nacional e internacional, a inserção da família no cuidado ao pré-termo ainda no contexto hospitalar proporciona benefícios relacionados ao desenvolvimento neuropsicomotor, ou seja, o contato mãe-bebê, através da pele, acelera a maturação cerebral, e a amamentação ajuda no aprimoramento cognitivo do pré-termo^(20,26)^.

Dessa forma, apesar das pontuações dos especialistas com relação ao item neuropsicomotor, em que a necessidade de manter tal temática foi questionada, não foram realizadas modificações no aplicativo, e todo o conteúdo foi mantido e justificado pela literatura disponível.

Além disso, o conteúdo do item estimulação neuropsicomotora, após avaliação do painel de especialistas, foi validado, o que reforça a relevância do tema para o público-alvo no domicílio, além da possibilidade de gerar benefícios ao pré-termo, como a redução de danos à saúde neonatal propiciados pela prematuridade.

Em adição, a interação entre usuário e aplicação, com a possibilidade de inserção de informações pelo próprio, foi criada com o intuito de proporcionar interesse àquele que a utiliza e fornecer material importante para o profissional da saúde nos casos de atendimentos futuros, como nas consultas de seguimento por meio da puericultura^(27)^.

Precipuamente, o aplicativo *Meu RN Prematuro* foi idealizado para ser um produto de utilidade materna fora da rede terciária, ou seja, um recurso para ser acessado após a alta hospitalar, com vistas a possibilitar a escolha da melhor conduta frente às questões relacionadas ao neonato e oferecer segurança nas tomadas de decisão no ambiente domiciliar.

No entanto, após a análise das respostas dos especialistas, notou-se que tal ferramenta vai muito além da simples disponibilização de conteúdo às mães de recém-nascidos pré-termo, mas sim constitui um importante recurso de educação em saúde para a área de saúde pública, devido ao seu alto grau de relevância para o cuidado.

Ademais, a possibilidade de acesso a materiais científicos, seja de forma física ou remota, pode contribuir para a resolução de questões relacionadas à saúde do pré-termo em diversas regiões do país, fornecendo subsídios para uma assistência centrada no usuário e em suas necessidades, evitando, assim, agravos à saúde.

### Limitações de estudo

Como limitações do presente estudo, considera-se o fato de a amostra de mães ter sido reduzida e poder não ser representativa do total de mães de crianças prematuras, além da avaliação semântica não ter sido contemplada nesta etapa da pesquisa. Ademais, o estudo com o envolvimento de outros profissionais responsáveis pelo cuidado neonatal poderia ter uma abrangência maior. No entanto, os resultados encontrados podem fomentar o interesse de pesquisadores em aprofundar a avaliação deste aplicativo, bem como investigar a utilidade desta ferramenta no cotidiano das mães de recém-nascidos prematuros.

### Contribuições para enfermagem e saúde

A criação de uma tecnologia desenvolvida com base em documentos científicos e avaliada por profissionais enfermeiros especialistas na temática, voltada para o cuidado ao pré-termo, não só é inovadora, como necessária, uma vez que nascimentos prematuros ainda ocorrem de forma preocupante. Além disso, manter a continuidade da assistência fora da rede hospitalar e reforçar a interrelação entre as redes de atenção à saúde é de extrema importância para a população brasileira, algo que pode ser possível por meio da utilização da ferramenta tecnológica.

## CONCLUSÕES

O estudo desenvolvido atingiu o objetivo proposto e revelou, por meio de especialistas na temática, graus de validação de conteúdo adequados, demonstrando que a tecnologia desenvolvida é válida, de fácil manuseio, com utilidade pública, inovadora e com conteúdo científico relevante e acessível.

Assim, tal ferramenta tecnológica, além da possibilidade de ser útil para mães e famílias de recém-nascidos pré-termo, constitui mais uma tecnologia de cuidado para o enfermeiro, uma vez que potencializa as ações de promoção e proteção à saúde da criança, por meio do cuidado direcionado e de orientações assertivas para o cuidador.

Ademais, como recomendação, sugere-se que, posteriormente, sejam realizados estudos de validação semântica e transcultural deste aplicativo móvel para apoio às mães de recém-nascidos pré-termo, visando à utilização dessa ferramenta em cenários internacionais.

## Data Availability

Não se aplica.
